# Prevalence of PD-1 Inhibitor-Associated Peripheral Neuropathy: A Retrospective Cohort Study

**DOI:** 10.3390/life16010031

**Published:** 2025-12-25

**Authors:** Suma Ganji-Angirekula, Nicole W. Segada, Prit Hasan, Peter M. Grace, Jian Wang, Xiaowen Sun, Saba Javed

**Affiliations:** 1 Department of Emergency Medicine, UTHealth Houston McGovern Medical School, Houston, TX 77030, USA; 2 Department of Anesthesiology, Baylor College of Medicine, Houston, TX 77030, USA; 3 Department of Medicine, Sam Houston State University College of Osteopathic Medicine, Conroe, TX 77304, USA; 4 Laboratories of Neuroimmunology, Department of Symptom Research, MD Anderson, Houston, TX 77030, USA; 5 Department of Biostatistics, MD Anderson, Houston, TX 77006, USA; 6 Department of Pain Medicine, Division of Anesthesiology, Critical Care Medicine, and Pain Medicine, MD Anderson, Houston, TX 77030, USA

**Keywords:** neuropathy, PD-1 inhibitor, immunotherapy

## Abstract

Immunotherapy is a promising treatment option for many cancers but is associated with the development of peripheral neuropathy in some patients. This retrospective cross-sectional EMR-based prevalence study was performed at MD Anderson Cancer Center with an aim to define the prevalence and epidemiology of Programmed Cell Death Protein 1 (PD-1) inhibitor-associated polyneuropathy. A total of 12,092 patients treated with a PD-1 inhibitor between 4 March 2016 and 18 June 2023 were identified and those on immunotherapy monotherapy were isolated. A total of 817 patients had documented neuropathy-associated with PD-1 inhibitor exposure, corresponding to a prevalence of 6.76% (6.76%, 95% CI 6.31–7.22). Data was stratified to assess for association between peripheral neuropathy and agent, sex, race, ethnicity, smoking and diabetes status. Patients identifying as “Other” race had higher prevalence of neuropathy compared to White or Caucasian patients (OR 1.514, *p* = 0.0189) and non-Hispanic or Latino patients had higher prevalence of neuropathy compared to Hispanic or Latino patients (OR 1.502, *p* = 0.0078). Current-smokers had significantly lower prevalence of neuropathy compared to never-smokers (OR 0.716, *p* = 0.0368). These disparities underscore the importance of further investigation in genetics and mechanisms to identify therapeutic interventions for PD-1 inhibitor-associated peripheral neuropathy.

## 1. Introduction

Immunotherapy has emerged as a groundbreaking approach in cancer treatment, harnessing the body’s immune system to combat malignancies. Typically, the immune system can recognize and inhibit cancer growth through a process termed immunosurveillance; however, cancer cells often develop sophisticated mechanisms to evade detection. These mechanisms include genetic mutations that make them less recognizable, the expression of surface proteins that deactivate immune cells and the alteration of surrounding cells to hinder immune responses. Immunotherapy aims to enhance the immune system’s capacity to identify, target and destroy cancer cells [1,2].

There are several types of immunotherapies, including checkpoint inhibitors, T cell transfer therapy, monoclonal antibodies, treatment vaccines and cytokine therapy. Checkpoint inhibitors are designed to block immune checkpoints that usually function to regulate immune responses, preventing overactivity. By inhibiting these checkpoints, the medications can amplify the immune system’s response against cancer [3]. Specifically, this study focuses on Programmed Cell Death Protein 1 (PD-1) inhibitors, including nivolumab, pembrolizumab, lambrolizumab and pidilizumab. These agents bind to the PD-1 receptor on T cells, preventing cancer cells from evading immune surveillance, thus enhancing T cell activity against tumors and promoting memory responses to tumor-specific antigens [4,5].

While the efficacy of these immunotherapies is well-documented, they are not without side effects. Common adverse effects include peripheral edema, skin rashes, gastrointestinal symptoms (nausea, vomiting, diarrhea), fatigue, liver injury, neutropenia, acid-base and electrolyte disturbances, endocrine disruption and more. Among these, peripheral neuropathy is particularly noteworthy [6,7]. Although chemotherapy-induced peripheral neuropathy (CIPN) has a well-established prevalence rate of approximately 30% [8,9,10], the prevalence and mechanisms of peripheral neuropathy specifically linked to checkpoint inhibitors have not been extensively studied. Although chemotherapy and immunotherapy share the potentially debilitating side effect of peripheral neuropathy, they differ in basic pathophysiology, presentation and management. While CIPN occurs via direct neurotoxicity with disruption of microtubules, mitochondrial damage and axonal degeneration [11], PD-1 inhibitor-associated peripheral neuropathy is thought to be driven by the therapy’s immunomodulating effects and can frequently lead to demyelination noted on electromyography (EMG) or nerve conduction studies (NCS) [12]. CIPN has a classic presentation of sensory neuropathy with numbness and paresthesia in a “glove and stocking” distribution, but PD-1 inhibitor-associated peripheral neuropathy has a wider spectrum of presentation ranging from headaches and dizziness to sensory or motor neuropathies to cranial neuropathies with meningitis and cranial nerve involvement or syndromes resembling Guillain–Barre or myasthenia gravis [13,14]. While PD-1 inhibitor-associated peripheral neuropathies have a wide range of presentations, they often present with progressive weakness, paresthesias, pain, gait instability and hyporeflexia or areflexia [12]. Mainstay of treatment for CIPN is largely supportive, whereas PD-1 inhibitor-associated peripheral neuropathies frequently also employ immunosuppression with agents like steroids or intravenous immunoglobulin (IVIG) in their management [6]. As a result of the relative novelty of immunotherapy, the existing literature primarily focuses on CIPN which highlights a gap in understanding regarding immunotherapy-related neuropathy.

Further characterization of the prevalence and epidemiology of PD-1 inhibitor-associated peripheral neuropathy would help guide patient management and optimize treatment strategies of this unfortunate side effect. We hypothesize that peripheral neuropathy may exhibit racial and ethnic disparities in its development and have higher prevalence in groups already at risk of developing neuropathy, such as smokers or patients with diabetes. This retrospective cross-sectional electronic medical record (EMR)-based prevalence study aims to define the prevalence of peripheral neuropathy in patients receiving PD-1 inhibitors and identify potential risk factors.

## 2. Materials and Methods

**Neuropathy identification (ICD-10):** After obtaining full Institutional Review Board (IRB) approval per MD Anderson Cancer Center policy, a retrospective cross-sectional EMR-based prevalence study was conducted. Medical records were obtained which included parameters such as patient demographics, diagnoses, smoking status, treatment regimens, presence of diabetes, body mass index (BMI) and presence of drug-induced neuropathy. We identified neuropathy events using prespecified ICD-10-CM codes recorded in the electronic medical record. Codes included G62.0 (Drug-induced polyneuropathy) and additional neuropathy codes used in clinical practice to capture treatment-related neuropathy presentations, including G62.2 (Polyneuropathy due to other toxic agents).

**Clinical confirmation criteria:** Neuropathy events were confirmed if documentation supported (1) new or worsening peripheral neuropathy symptoms (e.g., numbness/paresthesia, neuropathic pain, weakness), (2) clinician assessment consistent with peripheral neuropathy on exam and/or relevant diagnostic testing when available (EMG/NCS, neurology consultation) and (3) no clearly documented alternative primary cause (e.g., compressive radiculopathy alone, stroke, motor neuron disease). When attribution was explicitly documented, cases were classified as PD-1–related if the treating clinician (oncology/neurology) described the neuropathy as an immune-related adverse event, suspected drug-related toxicity, or recommended immune-toxicity management (e.g., corticosteroids, treatment hold).

**Manual chart validation:** All neuropathy cases identified through ICD-10 coding underwent manual chart review to confirm eligibility. Validation was performed by a clinician reviewer using a standardized abstraction template and prespecified criteria, including documentation of new-onset peripheral neuropathy symptoms, neurologic examination findings when available, temporal relationship to PD-1 inhibitor exposure (neuropathy diagnosis at least one month after initiation of PD-1 inhibitor therapy and within one year of initiation), clinician attribution and exclusion of alternative etiologies. Only patients on PD-1 inhibitor monotherapy between 4 March 2016 and 18 June 2023 were eligible for involvement in the study (combination therapies or concurrent chemotherapy were excluded). Reviewers were aware of PD-1 exposure status at the time of chart review. Formal inter-rater reliability assessment was not performed.

**Electronic medical record data extraction and validation:** Data were extracted from the electronic medical record using Epic SlicerDicer (Epic Systems, Verona, WI, USA), an institutional self-service reporting and cohort discovery tool that queries structured clinical data fields. PD-1 inhibitor exposure variables (agent, initiation date), neuropathy diagnoses (ICD-10 codes) and relevant clinical variables were obtained from structured encounter diagnoses, medication administration records and oncology treatment modules within Epic. To ensure data accuracy, extracted data were validated by two reviewers through targeted manual chart review. Reviewers confirmed key variables, including PD-1 exposure dates, timing of neuropathy diagnosis relative to treatment initiation and exclusion of alternative neuropathy etiologies or pre-existing disease. Discrepancies identified during validation were resolved by consensus review.

**Data analysis:** Original search criteria identified 36,567 patients who were prescribed one of the PD-1 inhibitors evaluated in this study (nivolumab, pembrolizumab, lambrolizumab and pidilizumab). Among the remaining 12,092 patients on immunotherapy monotherapy, a total of 817 patients were diagnosed with drug-induced polyneuropathy, and 11,275 patients were not diagnosed with drug-induced polyneuropathy. Patient demographics and clinical factors were summarized using descriptive statistics. Continuous variables were reported as mean, standard deviation (SD), median and range; categorical variables were reported as frequencies and proportions. Neuropathy prevalence with 95% confidence interval (CI) was provided. Factors were compared between patients with and without neuropathy using the Wilcoxon rank sum test for continuous variables and chi-square or Fisher’s exact test for categorical variables. Multivariable analysis was performed using logistic regression, with variance inflation factors (VIFs) assessed for multicollinearity. A complete-case analysis approach was used for the logistic regression model. Of the 12,092 patients in the study cohort, 11,860 (98.1%) had complete data and were included in the final analysis. Given the minimal proportion of missing data (<2%), no imputation was performed. A two-sided *p*-value of <0.05 was considered statistically significant. Analyses were conducted using SAS 9.4 (SAS Institute, Cary, NC, USA).

## 3. Results

**Cohort characteristics:** A total of 12,092 patients were identified. Our key findings included patients self-identifying as “Other” race (Table 1; Odds Ratio (OR) 1.514, 95% CI 1.071–2.140, *p* = 0.0189) and non-Hispanic or Latino ethnicity (Table 1; OR 1.502, 95% CI 1.113–2.028, *p* = 0.0078) having higher prevalence of documented neuropathy associated with PD-1 inhibitor exposure, and current-smokers having lower prevalence of documented neuropathy associated with PD-1 inhibitor exposure (Table 1; OR 0.716, 95% CI 0.523–0.980, *p* = 0.0368). Table 2 describes the continuous variables of our population, with an average age of 62.65 ± 13.67 (mean ± SD) and average BMI of 27.74 ± 6.92 (mean ± SD).

Table 3 examines the population by categorical variables including sex, race, ethnicity, smoking status, diabetes diagnosis and immunotherapy utilized. Our study population comprised 4815 (39.8%) females and 7277 (60.2%) males (Table 3). In terms of race and ethnicity, the majority of the group identified as White or Caucasian (9630 patients, 79.6%) and the majority identified as not Hispanic or Latino (10,403 patients, 86%) (Table 3). The majority of the patients reported some history of smoking, with 885 (7.4%) current-smokers and 5358 (44.9%) former smokers (Table 3). Diabetes diagnosis was found in 3022, or 25% of patients (Table 3). The most prevalent immunotherapy drug used was Pembrolizumab, utilized in 6999 (57.9%) of the patients (Table 3).

**Table 1:** Multivariable analysis for association between immunotherapy-induced neuropathy and demographic and clinical factors.

Variable	Odds Ratio (OR)	95% Confidence Intervals		*p*-Value
**Age**	0.996	0.991	1.002	0.1717
**BMI**	1.011	0.999	1.022	0.0715
**Sex: Female vs. Male**	1.069	0.921	1.240	0.3793
**Ethnic Group: Declined to Answer vs. Hispanic or Latino**	1.444	0.792	2.635	0.2305
**Ethnic Group: Non-Hispanic or Latino vs. Hispanic or Latino**	1.502	1.113	2.028	0.0078
**Primary Race: American Indian or Alaska vs. White or Caucasian**	0.588	0.142	2.431	0.4633
**Primary Race: Asian vs. White or Caucasian**	0.986	0.710	1.369	0.9335
**Primary Race: Black or African American vs. White or Caucasian**	1.229	0.952	1.585	0.1130
**Primary Race: Other vs. White or Caucasian**	1.514	1.071	2.140	0.0189
**Smoking Status: Yes vs. Never**	1.400	1.202	1.679	0.0272
**Smoking Status: Current vs. Never**	0.716	0.523	0.980	0.0368
**Diabetes: Yes vs. No**	1.116	0.945	1.320	0.1967
**Immunotherapy: pembrolizumab vs. nivolumab**	1.058	0.913	1.226	0.4554

**Table 2:** Patient characteristics and clinical outcomes (N = 12,092) were summarized by neuropathy status; Wilcoxon rank sum test was used to compare the patient characteristics and clinical outcomes between neuropathy and non-neuropathy for continuous variables.

	Neuropathy			
	No-Neuropathy (N = 11,275)	Neuropathy (N = 817)	Total (N = 12,092)	*p*-Value
**Age**				0.2860 ^1^
N (Missing)	11,275 (0)	817 (0)	12,092 (0)	
Mean (SD)	62.7 (13.69)	62.1 (13.28)	62.6 (13.67)	
Median	65.0	64.0	65.0	
Range	1.0, 98.0	16.0, 92.0	1.0, 98.0	
**BMI**				0.0577 ^1^
N (Missing)	11,216 (59)	810 (7)	12,026 (66)	
Mean (SD)	27.7 (6.26)	28.2 (6.70)	27.7 (6.29)	
Median	26.9	27.3	26.9	
Range	11.6, 99.3	13.8, 91.1	11.6, 99.3	

^1^ Wilcoxon rank sum *p*-value.

**Table 3:** Patient characteristics and clinical outcomes (N = 12,092) were summarized by neuropathy status, chi-square test or Fisher’s exact test were used to compare patient characteristics and clinical outcomes between neuropathy and non-neuropathy for categorical variables.

	Neuropathy			
	No-Neuropathy (N = 11,275)	Neuropathy (N = 817)	Total (N = 12,092)	*p*-Value
**Sex**, n (%)				0.2172 ^1^
Female	4473 (39.7%)	342 (41.9%)	4815 (39.8%)	
Male	6802 (60.3%)	475 (58.1%)	7277 (60.2%)	
**Primary Race**, n (%)				0.5043 ^2^
American Indian or Alaska Native	48 (0.4%)	2 (0.2%)	50 (0.4%)	
Asian	599 (5.3%)	43 (5.3%)	642 (5.3%)	
Black or African American	837 (7.4%)	76 (9.3%)	913 (7.6%)	
White or Caucasian	8995 (79.8%)	635 (77.7%)	9630 (79.6%)	
Native Hawaiian or Other Pacific Islander	12 (0.1%)	0 (0.0%)	12 (0.1%)	
Declined to Answer	66 (0.6%)	3 (0.4%)	69 (0.6%)	
Other	688 (6.1%)	57 (7.0%)	745 (6.2%)	
Unknown	30 (0.3%)	1 (0.1%)	31 (0.3%)	
**Ethnic Group**, n (%)				0.2923 ^1^
Declined to Answer	230 (2.0%)	16 (2.0%)	246 (2.0%)	
Hispanic or Latino	1291 (11.5%)	78 (9.5%)	1369 (11.3%)	
Not Hispanic or Latino	9687 (85.9%)	716 (87.6%)	10,403 (86.0%)	
Unknown	65 (0.6%)	7 (0.9%)	72 (0.6%)	
Missing	2	0	2	
**Smoking Status**, n (%)				0.0007 ^2^
Current	838 (7.5%)	47 (5.8%)	885 (7.4%)	
Former	4999 (45.0%)	359 (43.9%)	5358 (44.9%)	
Never	5109 (46.0%)	410 (50.2%)	5519 (46.3%)	
Passive Smoke Exposure—Never-Smoker	46 (0.4%)	0 (0.0%)	46 (0.4%)	
Unknown	119 (1.1%)	1 (0.1%)	120 (1.0%)	
Missing	164	0	164	
**Diabetes**, n (%)				0.1366 ^1^
No	8474 (75.2%)	592 (72.8%)	9066 (75.0%)	
Yes	2801 (24.8%)	221 (27.2%)	3022 (25.0%)	
Missing	0	4	4	
**Immunotherapy**, n (%)				0.4582 ^1^
Nivolumab	4759 (42.2%)	334 (40.9%)	5093 (42.1%)	
Pembrolizumab	6516 (57.8%)	483 (59.1%)	6999 (57.9%)	

^1^ Chi-Square *p*-value; ^2^ Fisher exact *p*-value.

**Neuropathy prevalence:** In our study, immunotherapy-associated neuropathy was identified in 817 patients, corresponding with a prevalence of 6.76% (6.76%, 95% CI 6.31–7.22).

**Multivariable analysis:** Table 1 reports the results for the association analysis between immunotherapy-associated neuropathy development and demographic and clinical factors, using multivariable analysis. When evaluating the association between immunotherapy and a neuropathy diagnosis, nivolumab and pembrolizumab were compared against each other. Of the patients who received immunotherapy and subsequently developed neuropathy, 40.9% received nivolumab and 59.1% received pembrolizumab (Table 3). When comparing pembrolizumab and nivolumab, there was no statistically significant difference in the documented neuropathy associated with PD-1 inhibitor exposure (Table 1; OR 1.058, 95% CI 0.913–1.226, *p* = 0.4554), after adjusting for other covariates. In examining race and ethnicity of the patients who had documented neuropathy associated with PD-1 inhibitor exposure, those who self-identified as “Other” race had a higher prevalence when compared to White or Caucasian patients (Table 1; OR 1.514, 95% CI 1.071–2.140, *p* = 0.0189) and those who identified as non-Hispanic or Latino had higher number of documented neuropathies associated with PD-1 inhibitor exposure when compared to Hispanic or Latino patients (Table 1; OR 1.502, 95% CI 1.113–2.028, *p* = 0.0078). When factoring in patients’ smoking history, there was a significantly lower prevalence of documented neuropathy associated with PD-1 inhibitor exposure in the current-smoker group when compared to the never-smoker group (Table 1; OR 0.716, 95% CI 0.523–0.980, *p* = 0.0368). Finally, we found that patients on immunotherapy with diabetes had a higher number of documented neuropathies associated with PD-1 inhibitor exposure compared to non-diabetic patients, although this finding was not statistically significant (Table 1; OR 1.116, 95% CI 0.945–1.320, *p* = 0.1967).

## 4. Discussion

Immunotherapy has gained popularity in the treatment options for cancer via mechanisms that use the body’s immune system to attack cancer cells. The efficacy of these treatments is well documented, and they have proven to improve outcomes in patients; however, the side effects of these medications, specifically the association with peripheral neuropathy development, has not been well studied in checkpoint inhibitors such as PD-1 inhibitors (including nivolumab, pembrolizumab, lambrolizumab and pidilizumab). Our retrospective cross-sectional EMR-based study identified a prevalence of 6.76% of the development of PD-1 inhibitor-associated polyneuropathy. Patients identifying as non-Hispanic Latino (when compared to Hispanic Latino patients) or “Other” race (when compared to White patients) had higher prevalence of PD-1 inhibitor-associated polyneuropathy. Patients identifying as current-smokers (when compared to never-smokers) had lower prevalence of PD-1 inhibitor-associated polyneuropathy. While there was increased prevalence of PD-1 inhibitor-associated polyneuropathy among diabetic patients, this finding did not reach statistical significance.

**Proposed mechanism of PD-1 inhibitor-associated neuropathy:** While PD-1 inhibitor upregulation of the immune system aids the recognition and elimination of cancer cells, the targets of the immune cells are unfortunately not limited to cancer cells. The precise pathogenesis of PD-1 inhibitor-associated neuropathy is not fully understood, and different mechanisms involving predisposition in a patient’s genetic profile or from prior viral infection have been postulated [15]. It has also been proposed that this same mechanism that lends PD-1 inhibitors’ efficacy in treatment of cancer may also predispose patients to neuropathy through autoreactivity against shared neural antigens which are both produced by tumor cells and present in peripheral nerves [15,16]. This phenomenon is amplified in patients with pre-existing autoimmune responses against neural antigens ectopically expressed by tumor cells prior to initiation of immunotherapy [15]. Furthermore, the inflammatory microenvironment produced by granzyme B and perforin-mediated apoptosis of cancerous and peripheral nerve tissues increases blood-brain barrier permeability and upregulates the expression of Major Histocompatibility Complex I (MHC I) on neurons [15]. Since MHC I displays antigens upon the cell surface, the immune response against peripheral nerves is potentiated, creating a positive feedback loop and leading to clinical symptoms of neuropathy. Additionally, endogenous PD-1/Programmed Cell Death Protein Ligand 1 (PD-L1) signaling produces antinociceptive effects in the peripheral nervous system via SHP-1 activation and TRPV1 inhibition in dorsal root ganglia [16,17,18]. Blocking this signaling with the initiation of immunotherapy further amplifies neuroinflammation and painful neuropathies. While risk factors for the development of PD-1 inhibitor-associated neuropathy have not yet been well established, studies such as ours have begun investigating patient demographics in hopes of identifying which patients are at increased risk. Combination immunotherapy [15,16], pre-existing autoimmunity [15,16] and certain types of cancer such as those associated with spontaneously occurring paraneoplastic neurological syndromes (i.e., lung or neuroendocrine malignancies) [15] seem to be implicated in development of PD-1 inhibitor-associated neuropathy. Other forms of neuropathy, such as CIPN, have common risk factors such as smoking status, diabetes history and predisposition in specific racial and ethnic groups. Additionally, although combination immunotherapy has been implicated in neuropathy development, it has not been well established if specific PD-1 inhibitors carry greater risk for neuropathy development. Our study aimed to further explore the prevalence and demographics of PD-1 inhibitor-associated neuropathy as it relates to the common risk factors of other neuropathies and compare different PD-1 inhibitors in their rates of PD-1 inhibitor-associated neuropathy.

**Racial and ethnic disparities in neuropathy development:** This study found that non-Hispanic Latino patients and those identifying as “Other” race experienced a higher prevalence of peripheral neuropathy when initiated on PD-1 inhibitors. While it is unclear which patient population is constituted by the “Other” race category, this patient selected identifier may capture patients of mixed racial backgrounds or minorities not adequately represented in standard race options. Although this trend does not imply causation or identify an underlying cause for preferential neuropathy development in this population, it is not the first to find racial or ethnic disparities in the development of neuropathy. When considering peripheral neuropathy as a whole, there have been conflicting findings with some studies establishing that non-Hispanic Black patients had lower odds of neuropathy development [19] while others demonstrate Black race having an association with higher prevalence of neuropathy [20]. However, these studies coincide on their finding that neuropathy is largely underdiagnosed, whether in non-Hispanic Black patients [19], or non-diabetic patients [20]. In patients with Human Immunodeficiency Virus (HIV), studies have illustrated that Black race is associated with higher prevalence of HIV-associated neuropathy [21], and African mitochondrial deoxyribonucleic acid (DNA) subhaplogroups were associated with nucleoside reverse transcriptase inhibitor-induced neuropathy in non-Hispanic Black patients [22]. Other studies have found that Hispanic ethnicity lends itself to higher prevalence of antiretroviral-associated neuropathy [23]. When considering diabetic neuropathy, studies have associated Caucasians and Europeans with higher prevalences of diabetic neuropathy when compared to their African-Caribbean and Indo-Asian counterparts. This is attributed to differences in skin microvascularization [24], and it is postulated that specific Vascular Endothelial Growth Factor (VEGF) genotypes may confer protection against ischemic neuronal damage and subsequent peripheral neuropathy development [25]. Examining chemotherapy, it is well established that Black patients and patients of non-European or African ancestry have higher prevalence of chemotherapy-induced peripheral neuropathy when compared to other races and ancestral backgrounds [26,27,28,29]. These findings were attributed to specific genotypic differences found among the patient groups [28,29] and underscores the potential benefit of genotyping to guide informed treatment decisions. Further study into the epidemiology of disease or medication-induced peripheral neuropathy is imperative to further establish trends, discern the mechanistic basis for disparities and identify actionable risk factors that can be addressed or considered when selecting treatment options for patients. Furthermore, many studies highlighted the underdiagnosis of peripheral neuropathy in multiple patient populations, which underscores the importance of standardized screening tools to allow early intervention.

**Neuropathy development in smokers:** Counterintuitively, while smoking is a known risk factor for neuropathic pain with a duration-dependent relationship [30,31,32], our study found that current-smokers had lower prevalence of PD-1 inhibitor-related neuropathy. Curiously, current smoking status has been demonstrated to impart protection against the development of conditions including ulcerative colitis (UC) and Parkinson’s disease [33,34,35,36]. This raises the question of the presence of a common mechanism for the potentially protective effects in these conditions and PD-1 inhibitor-related neuropathy. Studies postulate that nicotine’s protection against UC and Parkinson’s disease is attributable to its anti-inflammatory effects. Nicotine’s activation of nicotinic acetylcholine receptors (nAChRs) in the central and peripheral nervous system and muscle reduces inflammatory response and mitigates cognitive impairment and pain perception [36,37,38,39]. The activation of nAChRs influences a patient’s immune profile which may explain the lower prevalence of neuropathy in those receiving immunotherapy and provide a potential therapeutic target. Furthermore, current or former smokers have significantly higher response rates to PD-1 inhibitors when compared to never smokers, leading ultimately to increased survival and improved clinical outcomes [40,41,42]. While smoking leads to numerous health hazards and is not advised despite these findings, the anti-inflammatory and immune regulating effects of nicotine warrant further investigation. Pharmaceutical development targeting the beneficial effects of nicotine while omitting its deleterious properties may produce medications that can synergistically work with PD-1 inhibitors in combating cancer while additionally protecting against side effects such as neuropathic pain.

**Neuropathy development in diabetes:** The last key finding of our study noted that the prevalence of neuropathy development was higher in diabetic patients initiated on PD-1 inhibitors when compared to non-diabetic counterparts, although this difference did not reach statistical significance. Diabetes is an established and prevalent cause of peripheral neuropathy and is notorious for its contributions to worsening distal wounds, ultimately leading to infections and amputations. The pathophysiology behind diabetic neuropathy is not fully understood, but is thought to be multifactorial with contributions including hyperglycemia-induced vascular damage leading to neuronal hypoxic ischemia, hyperglycemia-activated pro-inflammatory pathways, impaired insulin function preventing neuronal repair and promoting neuronal death and neuronal damage secondary to advanced glycation end products and oxidative stress [43,44,45,46,47,48,49,50,51]. While diabetes predisposes patients to neuropathy development, immunotherapy may not contribute an additive effect. Alternatively, many patients with a prior diagnosis of diabetic neuropathy may not have been additionally diagnosed with immunotherapy-associated polyneuropathy due to difficulty distinguishing from their baseline sensory deficits. When considering multifactorial peripheral neuropathies, Gu et al. demonstrated through their meta-analysis that diabetic patients are at increased risk of chemotherapy-induced peripheral neuropathy [52]. While our dataset did not determine significance in the increased prevalence of peripheral neuropathy in diabetic patients initiated on PD-1 inhibitors, it remains possible that with further investigation a significant association may be established.

**Future research opportunities:** Knowledge of the effects (or potential effects) of epidemiology, smoking status and diabetes diagnosis on the prevalence of PD-1 inhibitor-associated neuropathy will allow patients to make informed decisions regarding their treatment choices. This guidance is invaluable as treatment options for neuropathies, including CIPN and immunotherapy-associated neuropathy, remain limited. Emerging therapies such as scrambler therapy, a noninvasive neuromodulation technique that uses paired electrodes to deliver electrical impulses in aim to rewire pain signals into non-painful sensations over time, show promise in the treatment of neuropathy [53]. Further investigation to establish causal relationship (if present) and the underlying mechanism between PD-1 inhibitor initiation and development of neuropathy may identify pharmaceutical targets for the prevention of immunotherapy-associated neuropathy and amplification of the efficacy of PD-1 inhibitors in the treatment of cancers.

**Limitations:** Similarly to other studies, ours has notable limitations. Our study’s retrospective design provides an inferior level of evidence compared to prospective studies. Retrospective studies are also limited in their ability to determine causation and therefore our findings are insufficient to establish a causal relationship between immunotherapy and neuropathy. Severity and length of neuropathy were not measured in this study; consequently, we are unable to evaluate how these variables may differ between the immunotherapy agents included in this study. Furthermore, the prevalence we found may possibly be an underestimation of true prevalence due to undercoding, potentially inadequately capturing mild neuropathy and differential screening practices across providers. A prospective study may be more useful to help develop a temporal relationship between immunotherapy treatment and development of neuropathy. Likewise, a longitudinal study may also provide more insight into long-term implications and management of patients on immunotherapy. As previously mentioned, it is possible that people may have a pre-existing diagnosis of diabetic neuropathy which precluded them from being labeled with the diagnosis of drug-induced polyneuropathy; this may have caused a discrepancy in coding diagnoses, preventing inclusion of patients with multifactorial diabetic and drug-induced neuropathy in our analysis. Inclusion of patients with multifactorial neuropathy may have elucidated a significant association between the presence of diabetes and the development of immunotherapy-induced peripheral neuropathy which was not captured in the current study. Similarly, other confounding variables which lead to pre-existing neuropathy prior to initiation of immunotherapies may not be adequately captured in the current study. Tumor type and stage were not analyzed in the current study, which in future investigations may highlight certain malignancies having higher predisposition for neuropathy development. Methodologic limitations of our study include the lack of blinding of reviewers to exposure status and lack of formal inter-rater reliability testing. Finally, our study was conducted at a single tertiary cancer center, which limits generalizability onto broader populations.

## 5. Conclusions

This study identified a prevalence of PD-1 inhibitor-associated neuropathy of 6.76%. Non-Hispanic Latino patients and those identifying as “Other” race experienced a higher prevalence of PD-1 inhibitor-associated neuropathy. This racial and ethnic disparity highlights a potential benefit in genetic analysis to inform patients of their risk and identify therapeutic targets. Curiously, our study found that current-smokers on immunotherapy had a lower prevalence of PD-1 inhibitor-associated neuropathy. Further research is needed to determine the reproducibility of this observation and establish its mechanism. The last key finding was that diabetes was not associated with an increased prevalence of immunotherapy-induced peripheral neuropathy, although our study was limited in the ability to capture patients with pre-existing diabetic neuropathy who may have developed a worsened multifactorial neuropathy upon initiation of immunotherapy. Peripheral neuropathy is a debilitating side effect of the increasingly utilized PD-1 inhibitors, and our findings underscore the importance of increased awareness and standardized screening practices for immunotherapy-associated neuropathy.

## Data Availability

The original contributions presented in this study are included in the article. Further inquiries can be directed to the corresponding author.

## Abbreviations

The following abbreviations are used in this manuscript:

PD-1	Programmed Cell Death Protein 1
PD-L1	Programmed Cell Death Protein Ligand 1
CIPN	Chemotherapy-Induced Peripheral Neuropathy
IVIG	Intravenous Immunoglobulin
EMR	Electronic Medical Record
IRB	Institutional Review Board
BMI	Body Mass Index
ICD	International Classification of Diseases
SD	Standard Deviation
CI	Confidence Interval
VIFs	Variance Inflation Factors
OR	Odds Ratio
HIV	Human Immunodeficiency Virus
DNA	Deoxyribonucleic Acid
VEGF	Vascular Endothelial Growth Factor
UC	Ulcerative Colitis
nAChRs	Nicotinic Acetylcholine Receptors
EMG	Electromyography
NCS	Nerve Conduction Studies
MHC I	Major Histocompatibility Complex I

## References

[1] Finn O.J. (2012). Immuno-oncology: Understanding the function and dysfunction of the immune system in cancer. Ann Oncol..

[2] Barbari C., Fontaine T., Parajuli P., Lamichhane N., Jakubski S., Lamichhane P., Deshmukh R.R. (2020). Immunotherapies and Combination Strategies for Immuno-Oncology. Int. J. Mol. Sci..

[3] Kumar A.R., Devan A.R., Nair B., Vinod B.S., Nath L.R. (2021). Harnessing the immune system against cancer: Current immunotherapy approaches and therapeutic targets. Mol. Biol. Rep..

[4] Boussiotis V.A. (2016). Molecular and Biochemical Aspects of the PD-1 Checkpoint Pathway. N. Engl. J. Med..

[5] Ai L., Xu A., Xu J. (2020). Roles of PD-1/PD-L1 Pathway: Signaling, Cancer, and Beyond. Adv. Exp. Med. Biol..

[6] Schneider B.J., Naidoo J., Santomasso B.D., Lacchetti C., Adkins S., Anadkat M., Atkins M.B., Brassil K.J., Caterino J.M., Chau I. (2021). Management of Immune-Related Adverse Events in Patients Treated with Immune Checkpoint Inhibitor Therapy: ASCO Guideline Update. J. Clin. Oncol..

[7] Petrelli F., Ardito R., Borgonovo K., Lonati V., Cabiddu M., Ghilardi M., Barni S. (2018). Haematological toxicities with immunotherapy in patients with cancer: A systematic review and meta-analysis. Eur. J. Cancer.

[8] Colvin L.A. (2019). Chemotherapy-induced peripheral neuropathy: Where are we now?. Pain.

[9] Seretny M., Currie G.L., Sena E.S., Ramnarine S., Grant R., MacLeod M.R., Colvin L.A., Fallon M. (2014). Incidence, prevalence, and predictors of chemotherapy-induced peripheral neuropathy: A systematic review and meta-analysis. Pain.

[10] Staff N.P., Grisold A., Grisold W., Windebank A.J. (2017). Chemotherapy-induced peripheral neuropathy: A current review. Ann. Neurol..

[11] Chen X., Gan Y., Au N.P.B., Ma C.H.E. (2024). Current understanding of the molecular mechanisms of chemotherapy-induced peripheral neuropathy. Front. Mol. Neurosci..

[12] Trimboli M., Marino L., Iusi I.C., Cucè M., Tozza S., Tagliaferri P., Tassone P., Gambardella A., Manganelli F., Liguori R. (2025). Polyradiculoneuropathies associated with immune checkpoint inhibitors: Are we facing a new nosological entity?. Neurol. Sci..

[13] Dubey D., David W.S., Amato A.A., Reynolds K.L., Clement N.F., Chute D.F., Cohen J.V., Lawrence D.P., Mooradian M.J., Sullivan R.J. (2019). Varied phenotypes and management of immune checkpoint inhibitor-associated neuropathies. Neurology.

[14] Tian Y., Gao A., Wen Q., Wang S., Zhang S., Yang X., Su G., Sun Y. (2020). Immune-Related Neurological Toxicities of PD-1/PD-L1 Inhibitors in Cancer Patients: A Systematic Review and Meta-Analysis. Front Immunol..

[15] Farina A., Villagrán-García M., Vogrig A., Zekeridou A., Muñiz-Castrillo S., Velasco R., Guidon A.C., Joubert B., Honnorat J. (2024). Neurological adverse events of immune checkpoint inhibitors and the development of paraneoplastic neurological syndromes. Lancet Neurol..

[16] Sarkar A., Nagappa M., Dey S., Mondal S., Babu G.S., Choudhury S.P., Akhil P., Debnath M. (2024). Synergistic effects of immune checkpoints and checkpoint inhibitors in inflammatory neuropathies: Implications and mechanisms. J. Peripher. Nerv. Syst..

[17] Liu B.L., Cao Q.L., Zhao X., Liu H.-Z., Zhang Y.-Q. (2020). Inhibition of TRPV1 by SHP-1 in nociceptive primary sensory neurons is critical in PD-L1 analgesia. JCI Insight.

[18] Wanderley C.W.S., Maganin A.G.M., Adjafre B., Mendes A.S., Silva C.E.A., Quadros A.U., Luiz J.P.M., Silva C.M.S., Silva N.R., Oliveira F.F.B. (2022). PD-1/PD-L1 Inhibition Enhances Chemotherapy-Induced Neuropathic Pain by Suppressing Neuroimmune Antinociceptive Signaling. Cancer Immunol. Res..

[19] Elafros M.A., Brown A., Marcus H., Dawood T., Bachuwa G.I., Banerjee M., Winch P.J., Kvalsund M., Feldman E.L., Skolarus L.E. (2024). Prevalence and Risk Factors of Distal Symmetric Polyneuropathy Among Predominantly Non-Hispanic Black, Low-Income Patients. Neurology.

[20] Hicks C.W., Wang D., Windham B.G., Matsushita K., Selvin E. (2021). Prevalence of peripheral neuropathy defined by monofilament insensitivity in middle-aged and older adults in two US cohorts. Sci Rep..

[21] Anziska Y., Helzner E.P., Crystal H., Glesby M.J., Plankey M., Weber K., Golub E., Burian P. (2012). The relationship between race and HIV-distal sensory polyneuropathy in a large cohort of US women. J. Neurol. Sci..

[22] Canter J.A., Robbins G.K., Selph D., Clifford D.B., Kallianpur A.R., Shafer R., Levy S., Murdock D.G., Ritchie M.D., Haas D.W. (2010). AIDS Clinical Trials Group Study 384 Team; New Work Concept Sheet 273 Team. African mitochondrial DNA subhaplogroups and peripheral neuropathy during antiretroviral therapy. J. Infect. Dis..

[23] Robinson-Papp J., Gonzalez-Duarte A., Simpson D.M., Rivera-Mindt M., Morgello S., Manhattan HIV Brain Bank (2009). The roles of ethnicity and antiretrovirals in HIV-associated polyneuropathy: A pilot study. J. Acquir. Immune Defic. Syndr..

[24] Abbott C.A., Chaturvedi N., Malik R.A., Salgami E., Yates A.P., Pemberton P.W., Boulton A.J. (2010). Explanations for the lower rates of diabetic neuropathy in Indian Asians versus Europeans. Diabetes Care.

[25] Zitouni K., Tinworth L., Earle K.A. (2017). Ethnic differences in the +405 and -460 vascular endothelial growth factor polymorphisms and peripheral neuropathy in patients with diabetes residing in a North London, community in the United Kingdom. BMC Neurol..

[26] Jordache P., Danahey K., Reizine N.M., O’Donnell P.H. (2021). Investigating the Prevalence and Risk of Chemotherapy-Induced Neuropathy Among Cancer Patients. J. Clin. Oncol..

[27] Sun L.F., Maples K.T., Hall K.H., Liu Y., Cao Y., Joseph N.S., Hofmeister C.C., Kaufman J.L., Dhodapkar M., Nooka A.K. (2023). Impact of Black Race on Peripheral Neuropathy in Patients With Newly Diagnosed Multiple Myeloma Receiving Bortezomib Induction. JCO Oncol. Pract..

[28] Nakshatri S., Dinh P.C., Feldman D.R., Hamilton R.J., Vaughn D.J., Fung C., Kollmannsberger C.K., A Huddart R., Martin N.E., Einhorn L.H. (2023). The Impact of Population Pharmacogenomics and Risk Allele Frequencies on Cisplatin-Induced Peripheral Sensory Neuropathy (PSN). J. Clin. Oncol..

[29] Hertz D.L., Roy S., Motsinger-Reif A.A., Drobish A., Clark L.S., McLeod H.L., Carey L.A., Dees E.C. (2013). CYP2C8*3 increases risk of neuropathy in breast cancer patients treated with paclitaxel. Ann. Oncol..

[30] Josiah D.T., Vincler M.A. (2006). Impact of chronic nicotine on the development and maintenance of neuropathic hypersensitivity in the rat. Psychopharmacology.

[31] De Vita M.J., Maisto S.A., Ansell E.B., Zale E.L., Ditre J.W. (2019). Pack-years of tobacco cigarette smoking as a predictor of spontaneous pain reporting and experimental pain reactivity. Exp. Clin. Psychopharmacol..

[32] Çelik S.B., Can H., Sözmen M.K., Şengezer T., Kaplan Y.C., Utlu G., Şener A., Yılmaz A.A., Aygün O. (2017). Evaluation of the neuropathic pain in the smokers. Agri.

[33] Zhang X., Zhu Z., Zhu L., Guan Y., Zhu Z., Liu B., Ren H., Yang X. (2025). Integrating Mendelian Randomization with Single-cell Sequencing Data Reveals the Causal Effect and Related Mechanisms of Smoking on Parkinson’s Disease. Nicotine Tob Res..

[34] Mappin-Kasirer B., Pan H., Lewington S., Kizza J., Gray R., Clarke R., Peto R. (2020). Tobacco smoking and the risk of Parkinson disease: A 65-year follow-up of 30,000 male British doctors. Neurology.

[35] Mahid S.S., Minor K.S., Soto R.E., Hornung C.A., Galandiuk S. (2006). Smoking and inflammatory bowel disease: A meta-analysis. Mayo Clin. Proc..

[36] Lakhan S.E., Kirchgessner A. (2011). Anti-inflammatory effects of nicotine in obesity and ulcerative colitis. J. Transl. Med..

[37] Zhang W., Lin H., Zou M., Yuan Q., Huang Z., Pan X., Zhang W. (2022). Nicotine in Inflammatory Diseases: Anti-Inflammatory and Pro-Inflammatory Effects. Front. Immunol..

[38] Lu B., Kwan K., Levine Y.A., Olofsson P.S., Yang H., Li J., Joshi S., Wang H., Andersson U., Chavan S.S. (2014). α7 nicotinic acetylcholine receptor signaling inhibits inflammasome activation by preventing mitochondrial DNA release. Mol. Med..

[39] Hurst R., Rollema H., Bertrand D. (2013). Nicotinic acetylcholine receptors: From basic science to therapeutics. Pharmacol. Ther..

[40] Wang X., Ricciuti B., Alessi J.V., Nguyen T., Awad M.M., Lin X., E Johnson B., Christiani D.C. (2021). Smoking History as a Potential Predictor of Immune Checkpoint Inhibitor Efficacy in Metastatic Non-Small Cell Lung Cancer. J. Natl. Cancer Inst..

[41] Zhao W., Jiang W., Wang H., He J., Su C., Yu Q. (2021). Impact of Smoking History on Response to Immunotherapy in Non-Small-Cell Lung Cancer: A Systematic Review and Meta-Analysis. Front. Oncol..

[42] Li J.J.N., Karim K., Sung M., Le L.W., Lau S.C., Sacher A., Leighl N.B. (2020). Tobacco exposure and immunotherapy response in PD-L1 positive lung cancer patients. Lung Cancer.

[43] Greene D.A., Sima A.A., Stevens M.J., Feldman E.L., Lattimer S.A. (1992). Complications: Neuropathy, pathogenetic considerations. Diabetes Care.

[44] Yagihashi S., Yamagishi S., Wada R. (2007). Pathology and pathogenetic mechanisms of diabetic neuropathy: Correlation with clinical signs and symptoms. Diabetes Res. Clin. Pract..

[45] Albers J.W., Pop-Busui R. (2014). Diabetic neuropathy: Mechanisms, emerging treatments, and subtypes. Curr. Neurol. Neurosci. Rep..

[46] Wada R., Yagihashi S. (2005). Role of advanced glycation end products and their receptors in development of diabetic neuropathy. Ann. N. Y. Acad. Sci..

[47] Vincent A.M., Russell J.W., Low P., Feldman E.L. (2004). Oxidative stress in the pathogenesis of diabetic neuropathy. Endocr. Rev..

[48] Van Dam P.S., Cotter M.A., Bravenboer B., Cameron N.E. (2013). Pathogenesis of diabetic neuropathy: Focus on neurovascular mechanisms. Eur. J. Pharmacol..

[49] Cameron N.E., Eaton S.E., Cotter M.A., Tesfaye S. (2001). Vascular factors and metabolic interactions in the pathogenesis of diabetic neuropathy. Diabetologia.

[50] Rajchgot T., Thomas S.C., Wang J.C., Ahmadi M., Balood M., Crosson T., Dias J.P., Couture R., Claing A., Talbot S. (2019). Neurons and Microglia; A Sickly-Sweet Duo in Diabetic Pain Neuropathy. Front. Neurosci..

[51] Song J., Kang S.M., Kim E., Kim C.H., Song H.T., Lee J.E. (2015). Impairment of insulin receptor substrate 1 signaling by insulin resistance inhibits neurite outgrowth and aggravates neuronal cell death. Neuroscience.

[52] Gu J., Lu H., Chen C., Gu Z., Hu M., Liu L., Yu J., Wei G., Huo J. (2021). Diabetes mellitus as a risk factor for chemotherapy-induced peripheral neuropathy: A meta-analysis. Support Care Cancer.

[53] Childs D.S., Le-Rademacher J.G., McMurray R., Bendel M., O’Neill C., Smith T.J., Loprinzi C.L. (2021). Randomized Trial of Scrambler Therapy for Chemotherapy-Induced Peripheral Neuropathy: Crossover Analysis. J. Pain Symptom Manag..

